# Supplementary material for: Modelling black carbon absorption of solar radiation: combining external and internal mixing assumptions
Source: Atmos Chem Phys. Author manuscript; Available in PMC 2020 Jan 7. (PMC6392454; doi:10.5194/acp-19-181-2019)
Supplement: Supplement1 [file NIHMS1519935-supplement-Supplement1.pdf]

Supplement of Atmos. Chem. Phys., 19, 181–204, 2019
https://doi.org/10.5194/acp-19-181-2019-supplement
© Author(s) 2019. This work is distributed under
the Creative Commons Attribution 4.0 License.

Atmospheric Chemistry and Physics
Open Access

Supplement of

Modelling black carbon absorption of solar radiation: combining external and internal mixing assumptions

Gabriele Curci et al.

Correspondence to: Gabriele Curci (gabriele.curci@aquila.infn.it)

The copyright of individual parts of the supplement might differ from the CC BY 4.0 License.

SUPPLEMENTARY ONLINE MATERIAL (SOM)

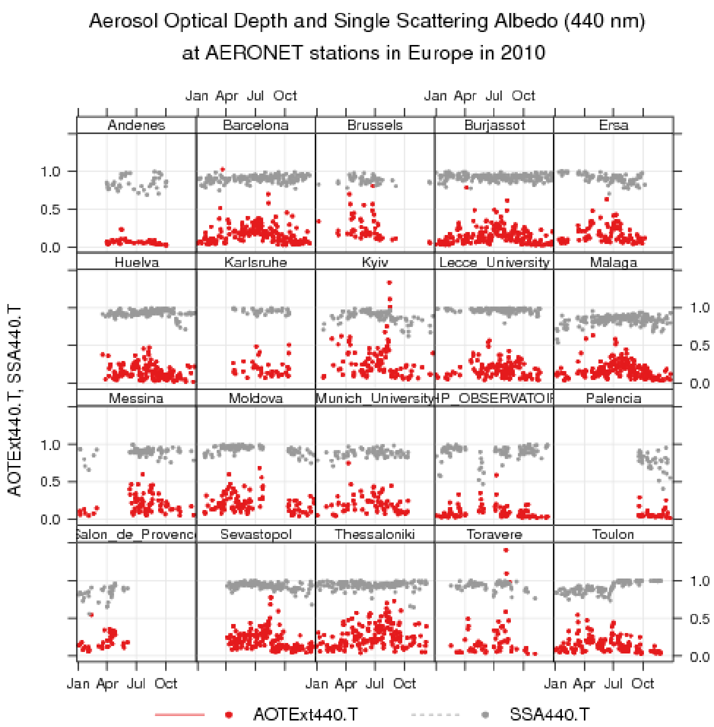

Figure S 1. Time series of aerosol optical depth and single scattering albedo at 440 nm observed over Europe in 2010 at AERONET stations selected in this study (see main paper for details on selection)

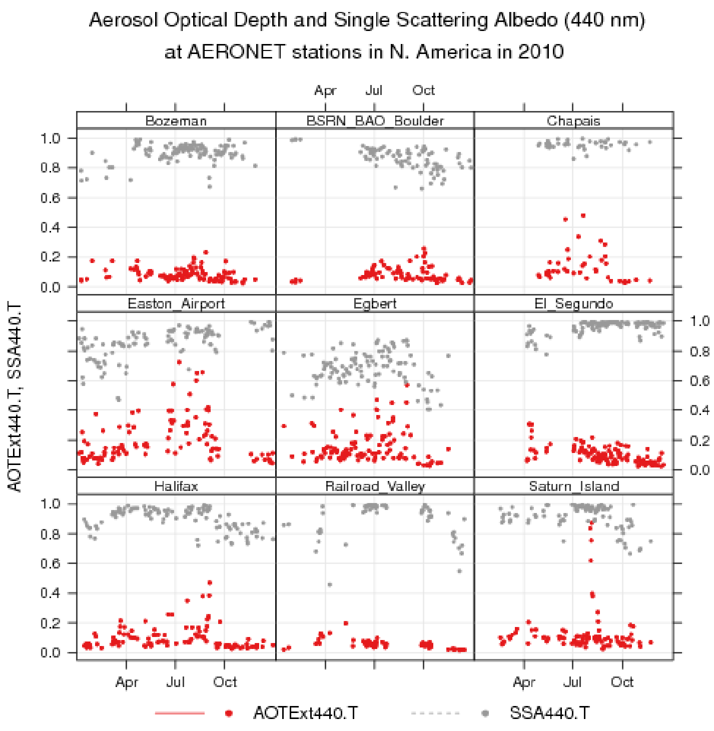

Figure S 2. Same as Figure S 1, but for North America

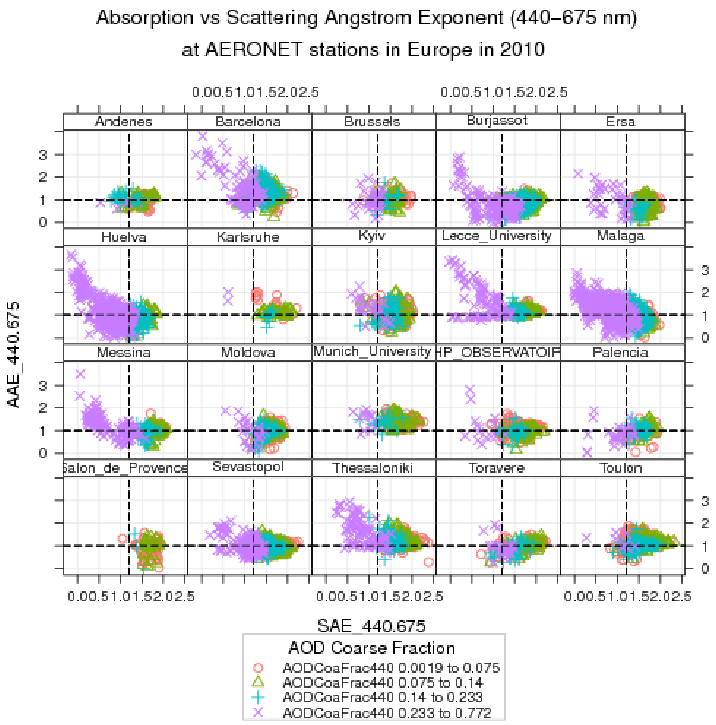

Figure S 3. Scatterplot of absorption Angstrom exponent (AAE) vs. scattering Angstrom exponent (SAE) between 440 and 675 nm at AERONET sites selected over Europe. Scenes having a SAE ≤ 1.2 are labelled as "Dust"-dominated, those having SAE > 1.2 and AAE < 1.2 as "BC"-dominated, and the remaining as "BC+BrC"-dominated. Data are coloured according to coarse fraction classes, as denoted by the legend inset.

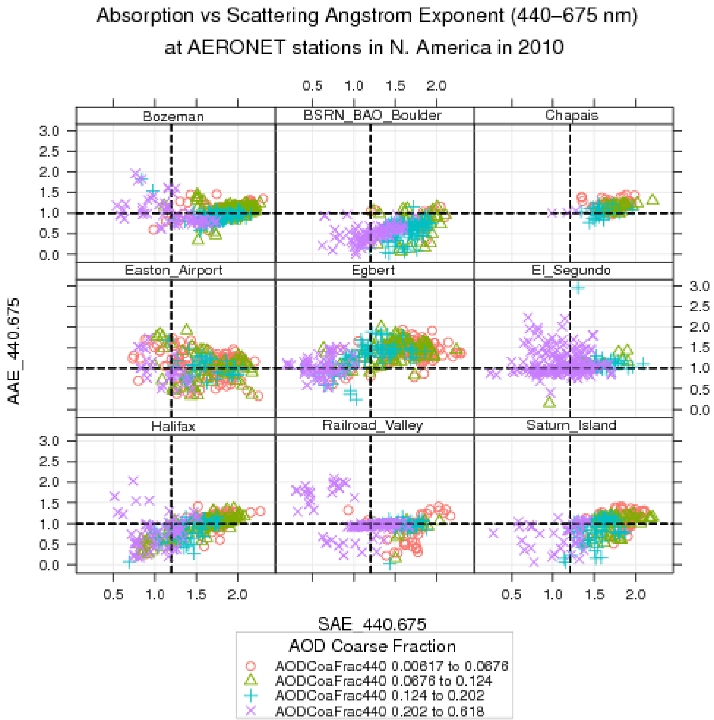

Figure S 4. Same as Figure S 3, but for North America.

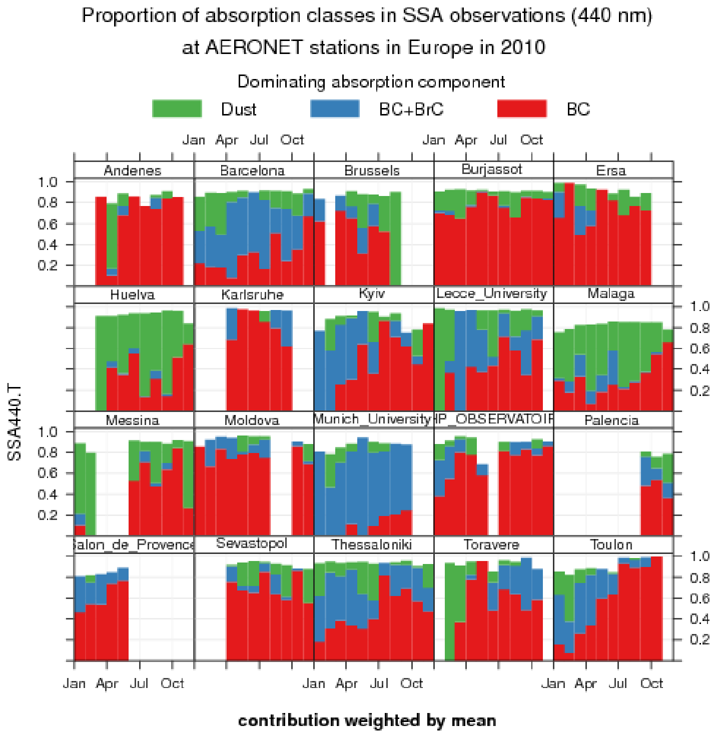

Figure S 5. Relative abundance of absorption classes in monthly mean single scattering albedo at AERONET sites in Europe.

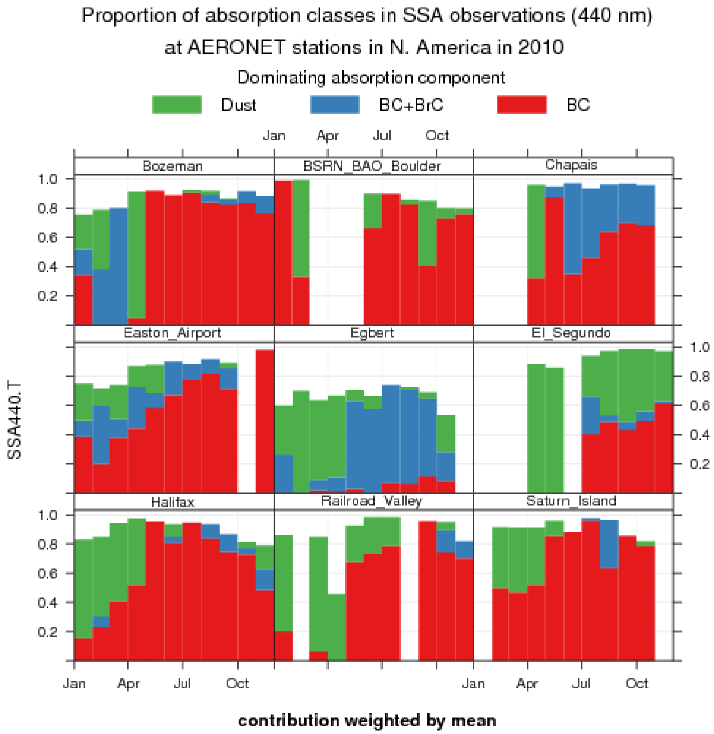

Figure S 6. Same as Figure S 5, but for North America.

~~**Table S 1. Mean PM2.5 concentration (µg/m^3) and aerosol optical depth at 555 nm (τ_{555}) calculated by different regional air quality models for year 2010 at available surface stations over Europe and North America. FRES1 model data are not available for these variables. Aerosol optical depths are not calculated with FlexAOD, but by each group with specific assumptions and methods.**~~

Table S 1. Mean PM2.5 concentration (µg/m^3), aerosol optical depth at 555 nm (τ_{555}) and PM composition (µg/m^3) calculated by different regional air quality models for year 2010 at available surface stations over Europe and North America. Aerosol optical depths are not calculated with FlexAOD, but by each group with specific assumptions and methods.

	PM2.5	τ_{555}	SO_4	NO_3	NH_4	OC	BC
Europe							
Observation	15.1	0.19	0.86	0.47	0.44	1.40	0.28
DE1	6.3	-	1.24	1.76	0.93	0.35	0.34
DK1	9.3	-	1.68	1.32	1.01	0.81	0.45
ES1	15.5	0.34	0.61	2.59	0.98	0.03	0.41
FI1	11.4	0.16	2.63	1.16	0.98	3.80	1.10
FRES1	-	-	2.23	1.18	0.79	1.18	0.70
IT2	8.6	0.16	2.22	0.68	0.99	1.33	0.54
NL1	7.5	-	1.37	1.39	0.91	1.45	0.73
TR1	13.3	-	2.00	4.11	2.00	1.43	1.29
UK3	9.3	-	1.94	1.56	1.13	1.04	0.70
N. America							
Observation	8.7	0.100	1.09	0.49	0.55	0.92	0.28
DK1	6.3	0.097	1.90	0.75	0.86	0.67	0.30
US3	8.6	0.098	1.10	1.01	0.61	1.15	0.50

Formattato ...
Formattato ...
Tabella formattata ...
Formattato ...
Formattato ...
Formattato ...
Formattato ...
Formattato ...
Formattato ...
Formattato ...
Formattato ...
Formattato ...
Tabella formattata ...
Formattato ...
Formattato ...
Formattato ...
Formattato ...
Formattato ...
Formattato ...
Formattato ...
Formattato ...
Formattato ...
Formattato ...
Formattato ...
Formattato ...
Formattato ...
Formattato ...
Formattato ...
Formattato ...
Formattato ...
Formattato ...
Formattato ...
Formattato ...
Formattato ...
Formattato ...
Formattato ...
Formattato ...
Formattato ...
Formattato ...
Formattato ...
Formattato ...
Formattato ...
Formattato ...
Formattato ...
Formattato ...
Formattato ...
Formattato ...
Formattato ...
Formattato ...
Formattato ...
Formattato ...
Formattato ...
Formattato ...

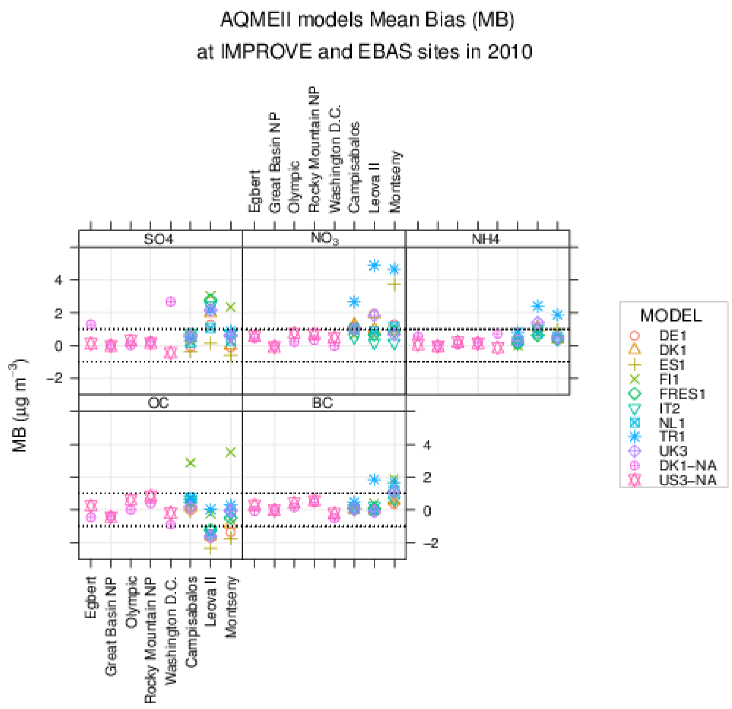

Figure S 7. Mean bias of PM composition ($\mu g/m^3$) calculated by different regional air quality models for year 2010 at available surface stations over Europe (3 sites, EMEP network/EBAS database) and North America (5 sites, IMPROVE network). The dashed horizontal lines denote ±1 $\mu g/m^3$ for qualitative guidance.

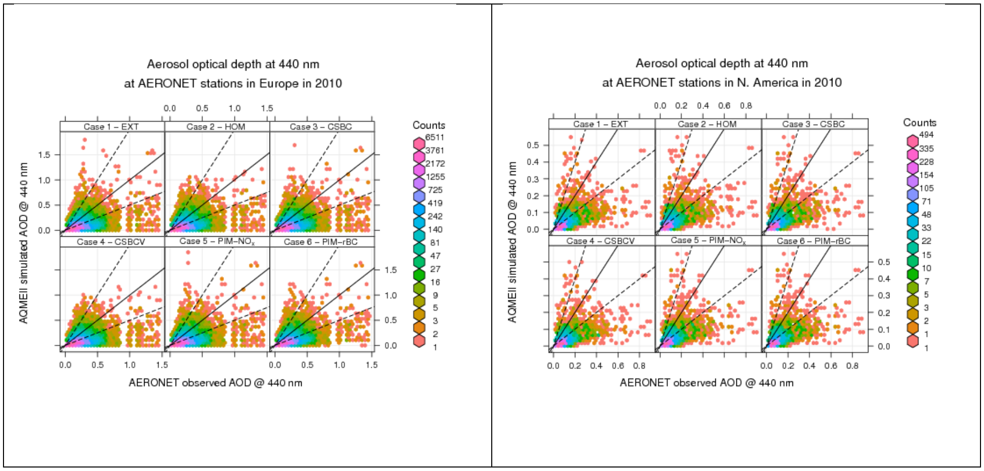

Figure S 87. Comparison of modelled and observed aerosol optical depths at 440 nm (τ_{440}) in 2010 at AERONET stations over Europe and North America, only for "BC" and "BC+BrC"-dominated scenes (see Table 1). Simulation labels are defined in Table 5.

Table S 22. Comparison of modelled and observed aerosol optical depths at 440 nm (τ_{440}) in 2010 at AERONET stations over Europe and North America, only for "BC" and "BC+BrC"-dominated scenes (see Table 1). Simulation labels are defined in Table 5 and statistical indices are defined in the Appendix. The number of data *n* may vary from case to case, due to numerical failures in the optical calculations.

Europe	*n*	$\bar{O}$	$\bar{M}$	σ_O	σ_M	*FAC2*	*MB*	*NMB*	*RMSE*	*r*
1.EXT	48911	0.18	0.07	0.14	0.09	0.27	-0.11	-0.59	0.16	0.50
2.HOM	48889	0.18	0.08	0.14	0.09	0.29	-0.10	-0.57	0.16	0.50
3.CSBC	48904	0.18	0.08	0.14	0.09	0.28	-0.10	-0.58	0.16	0.50
4.CSBCV	48891	0.18	0.07	0.14	0.08	0.24	-0.11	-0.62	0.17	0.49
5.PIM-NOx	48907	0.18	0.08	0.14	0.09	0.28	-0.11	-0.58	0.16	0.50
6.PIM-rBC	37999	0.19	0.08	0.14	0.10	0.30	-0.11	-0.56	0.16	0.52
N. America	*n*	$\bar{O}$	$\bar{M}$	σ_O	σ_M	*FAC2*	*MB*	*NMB*	*RMSE*	*r*
1.EXT	4030	0.12	0.05	0.10	0.06	0.25	-0.07	-0.59	0.11	0.53
2.HOM	4030	0.12	0.05	0.10	0.06	0.27	-0.07	-0.57	0.11	0.54
3.CSBC	4030	0.12	0.05	0.10	0.06	0.25	-0.07	-0.59	0.11	0.54
4.CSBCV	4030	0.12	0.04	0.10	0.05	0.21	-0.07	-0.62	0.11	0.53
5.PIM-NOx	4030	0.12	0.05	0.10	0.06	0.25	-0.07	-0.59	0.11	0.53
6.PIM-rBC	3540	0.12	0.05	0.11	0.06	0.27	-0.07	-0.58	0.12	0.52

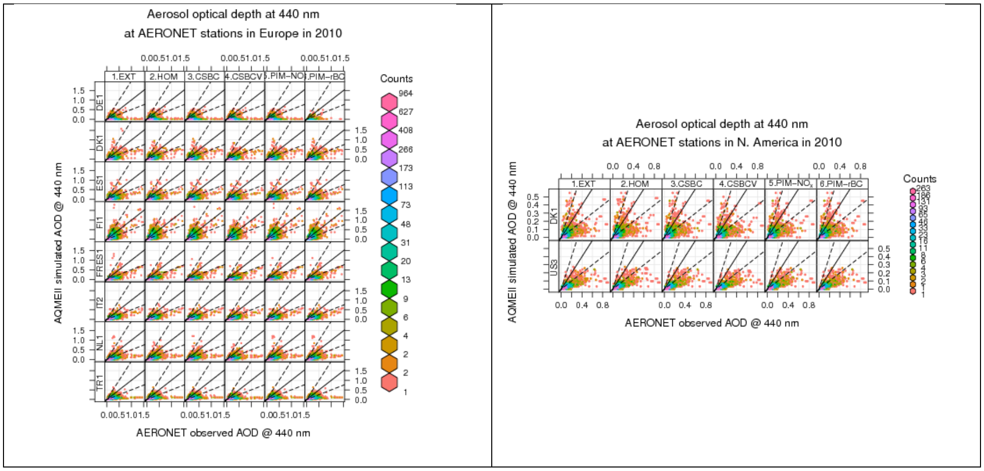

Figure S 98. Comparison of modelled and observed aerosol optical depths at 440 nm (τ_{440}) in 2010 at AERONET stations over Europe and North America, only for "BC" and "BC+BrC"-dominated scenes (see Table 1). Simulation labels are defined in Table 5. Unlike Figure S 8Figure S 7, the data is broken down by model.

Table S 33. Comparison of modelled and observed aerosol optical depths at 440 nm (τ_{440}) in 2010 at AERONET stations over Europe and North America, only for "BC" and "BC+BrC"-dominated scenes (see Table 1). Unlike Table S 2, the data is broken down by model. Simulation labels are defined in Table 5 and statistical indices are defined in the Appendix. The number of data *n* may vary from case to case, due to numerical failures in the optical calculations.

Label	Model	n	$\bar{O}$	$\bar{M}$	σ_O	σ_M	FAC2	MB	NMB	RMSE	r
Europe											
1.EXT	DE1	6115	0.18	0.04	0.14	0.06	0.14	-0.14	-0.77	0.20	0.22
1.EXT	DK1	6115	0.18	0.07	0.14	0.09	0.24	-0.11	-0.62	0.16	0.54
1.EXT	ES1	6115	0.18	0.07	0.14	0.08	0.25	-0.11	-0.61	0.16	0.52
1.EXT	FI1	6110	0.18	0.13	0.14	0.16	0.49	-0.05	-0.26	0.13	0.65
1.EXT	FRES1	6111	0.18	0.06	0.14	0.07	0.15	-0.12	-0.69	0.17	0.61
1.EXT	IT2	6115	0.18	0.07	0.14	0.07	0.27	-0.11	-0.59	0.15	0.70
1.EXT	NL1	6115	0.18	0.07	0.14	0.08	0.25	-0.11	-0.62	0.16	0.50
1.EXT	TR1	6115	0.18	0.07	0.14	0.06	0.36	-0.11	-0.59	0.16	0.45
2.HOM	DE1	6115	0.18	0.04	0.14	0.06	0.15	-0.14	-0.76	0.19	0.22
2.HOM	DK1	6111	0.18	0.07	0.14	0.08	0.27	-0.11	-0.60	0.16	0.56
2.HOM	ES1	6115	0.18	0.07	0.14	0.09	0.26	-0.11	-0.59	0.16	0.52
2.HOM	FI1	6099	0.18	0.14	0.13	0.15	0.51	-0.04	-0.23	0.13	0.63
2.HOM	FRES1	6108	0.18	0.06	0.14	0.07	0.17	-0.12	-0.67	0.16	0.65
2.HOM	IT2	6115	0.18	0.08	0.14	0.07	0.30	-0.10	-0.57	0.14	0.70

Label	Model	n	$\bar{O}$	$\bar{M}$	σ_O	σ_M	FAC2	MB	NMB	RMSE	r
2.HOM	NL1	6111	0.18	0.07	0.14	0.08	0.26	-0.11	-0.61	0.16	0.54
2.HOM	TR1	6115	0.18	0.08	0.14	0.06	0.39	-0.10	-0.56	0.16	0.46
3.CSBC	DE1	6115	0.18	0.04	0.14	0.06	0.15	-0.14	-0.77	0.20	0.22
3.CSBC	DK1	6113	0.18	0.07	0.14	0.08	0.25	-0.11	-0.61	0.16	0.55
3.CSBC	ES1	6115	0.18	0.07	0.14	0.09	0.26	-0.11	-0.60	0.16	0.52
3.CSBC	FI1	6109	0.18	0.14	0.14	0.16	0.50	-0.04	-0.24	0.13	0.65
3.CSBC	FRES1	6109	0.18	0.06	0.14	0.07	0.16	-0.12	-0.68	0.16	0.64
3.CSBC	IT2	6115	0.18	0.08	0.14	0.07	0.28	-0.11	-0.58	0.15	0.70
3.CSBC	NL1	6113	0.18	0.07	0.14	0.08	0.25	-0.11	-0.61	0.16	0.52
3.CSBC	TR1	6115	0.18	0.08	0.14	0.07	0.41	-0.10	-0.54	0.16	0.48
4.CSBCV	DE1	6115	0.18	0.04	0.14	0.05	0.13	-0.14	-0.78	0.20	0.21
4.CSBCV	DK1	6111	0.18	0.06	0.14	0.08	0.21	-0.12	-0.65	0.16	0.55
4.CSBCV	ES1	6115	0.18	0.07	0.14	0.08	0.24	-0.11	-0.63	0.17	0.51
4.CSBCV	FI1	6101	0.18	0.13	0.13	0.14	0.48	-0.05	-0.28	0.13	0.63
4.CSBCV	FRES1	6108	0.18	0.05	0.14	0.06	0.12	-0.13	-0.71	0.17	0.65
4.CSBCV	IT2	6115	0.18	0.07	0.14	0.07	0.22	-0.11	-0.62	0.15	0.70
4.CSBCV	NL1	6111	0.18	0.06	0.14	0.07	0.21	-0.12	-0.65	0.17	0.53
4.CSBCV	TR1	6115	0.18	0.07	0.14	0.06	0.30	-0.11	-0.63	0.17	0.44
5.PIM-NOx	DE1	6115	0.18	0.04	0.14	0.06	0.15	-0.14	-0.77	0.20	0.22
5.PIM-NOx	DK1	6113	0.18	0.07	0.14	0.08	0.25	-0.11	-0.61	0.16	0.55
5.PIM-NOx	ES1	6115	0.18	0.07	0.14	0.09	0.26	-0.11	-0.60	0.16	0.52
5.PIM-NOx	FI1	6110	0.18	0.14	0.14	0.16	0.50	-0.04	-0.24	0.13	0.65
5.PIM-NOx	FRES1	6109	0.18	0.06	0.14	0.07	0.15	-0.12	-0.68	0.16	0.64
5.PIM-NOx	IT2	6115	0.18	0.08	0.14	0.07	0.27	-0.11	-0.58	0.15	0.70
5.PIM-NOx	NL1	6115	0.18	0.07	0.14	0.08	0.25	-0.11	-0.61	0.16	0.51
5.PIM-NOx	TR1	6115	0.18	0.08	0.14	0.06	0.40	-0.10	-0.55	0.16	0.47
6.PIM-rBC	DE1	662	0.23	0.05	0.14	0.07	0.13	-0.18	-0.78	0.23	0.17
6.PIM-rBC	DK1	5989	0.18	0.07	0.14	0.08	0.25	-0.11	-0.62	0.16	0.55
6.PIM-rBC	ES1	4290	0.20	0.08	0.15	0.09	0.26	-0.12	-0.61	0.18	0.50
6.PIM-rBC	FI1	4457	0.20	0.16	0.15	0.17	0.56	-0.04	-0.19	0.14	0.63
6.PIM-rBC	FRES1	6108	0.18	0.06	0.14	0.07	0.15	-0.12	-0.68	0.16	0.63
6.PIM-rBC	IT2	5292	0.19	0.08	0.14	0.07	0.30	-0.11	-0.58	0.15	0.69
6.PIM-rBC	NL1	5618	0.19	0.07	0.14	0.08	0.26	-0.12	-0.61	0.17	0.49
6.PIM-rBC	TR1	5583	0.19	0.08	0.14	0.06	0.39	-0.11	-0.56	0.16	0.45
N. America											
1.EXT	DK1	2016	0.12	0.06	0.10	0.07	0.33	-0.06	-0.51	0.11	0.47

Label	Model	n	$\bar{O}$	$\bar{M}$	σ_O	σ_M	FAC2	MB	NMB	RMSE	r
1.EXT	US3	2014	0.12	0.04	0.10	0.04	0.16	-0.08	-0.67	0.11	0.72
2.HOM	DK1	2016	0.12	0.06	0.10	0.07	0.35	-0.06	-0.48	0.11	0.47
2.HOM	US3	2014	0.12	0.04	0.10	0.04	0.19	-0.08	-0.66	0.11	0.72
3.CSBC	DK1	2016	0.12	0.06	0.10	0.07	0.34	-0.06	-0.50	0.11	0.47
3.CSBC	US3	2014	0.12	0.04	0.10	0.04	0.17	-0.08	-0.67	0.11	0.73
4.CSBCV	DK1	2016	0.12	0.05	0.10	0.06	0.29	-0.06	-0.55	0.11	0.46
4.CSBCV	US3	2014	0.12	0.03	0.10	0.04	0.14	-0.08	-0.70	0.12	0.72
5.PIM-NOx	DK1	2016	0.12	0.06	0.10	0.07	0.34	-0.06	-0.50	0.11	0.47
5.PIM-NOx	US3	2014	0.12	0.04	0.10	0.04	0.17	-0.08	-0.67	0.11	0.72
6.PIM-rBC	DK1	1974	0.12	0.06	0.11	0.07	0.34	-0.06	-0.50	0.11	0.47
6.PIM-rBC	US3	1566	0.13	0.04	0.11	0.04	0.17	-0.09	-0.67	0.12	0.71

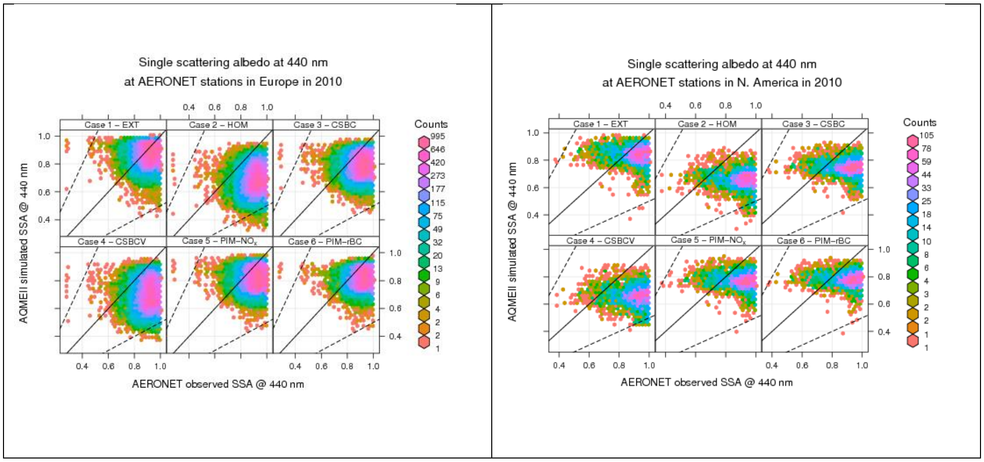

Figure S 109. Same as Figure S 8Figure S 7, but for single scattering albedo at 440 nm ($\omega_{0,440}$).

Table S 44. Same as Table S 1, but for single scattering albedo at 440 nm ($\omega_{0,440}$).

Europe	*n*	$\bar{O}$	$\bar{M}$	σ_O	σ_M	*FAC2*	*MB*	*NMB*	*RMSE*	*r*
1.EXT	48911	0.91	0.87	0.07	0.07	1.00	-0.04	-0.04	0.10	0.05
2.HOM	48889	0.91	0.69	0.07	0.09	0.99	-0.22	-0.24	0.24	0.06
3.CSBC	48904	0.91	0.79	0.07	0.07	1.00	-0.12	-0.13	0.15	0.04
4.CSBCV	48891	0.91	0.70	0.07	0.09	1.00	-0.21	-0.23	0.23	0.04
5.PIM-NOx	47498	0.91	0.82	0.06	0.07	1.00	-0.09	-0.10	0.13	0.03
6.PIM-rBC	37723	0.91	0.82	0.06	0.06	1.00	-0.09	-0.10	0.13	0.06
N. America	*n*	$\bar{O}$	$\bar{M}$	σ_O	σ_M	*FAC2*	*MB*	*NMB*	*RMSE*	*r*
1.EXT	4030	0.88	0.84	0.10	0.06	1.00	-0.04	-0.05	0.13	-0.11
2.HOM	4030	0.88	0.66	0.10	0.07	0.98	-0.22	-0.25	0.26	-0.13
3.CSBC	4030	0.88	0.75	0.10	0.06	1.00	-0.13	-0.15	0.18	-0.12
4.CSBCV	4030	0.88	0.66	0.10	0.07	0.99	-0.22	-0.25	0.26	-0.12
5.PIM-NOx	4029	0.88	0.77	0.10	0.06	1.00	-0.11	-0.12	0.16	-0.13
6.PIM-rBC	3540	0.88	0.78	0.10	0.05	1.00	-0.10	-0.11	0.16	-0.11

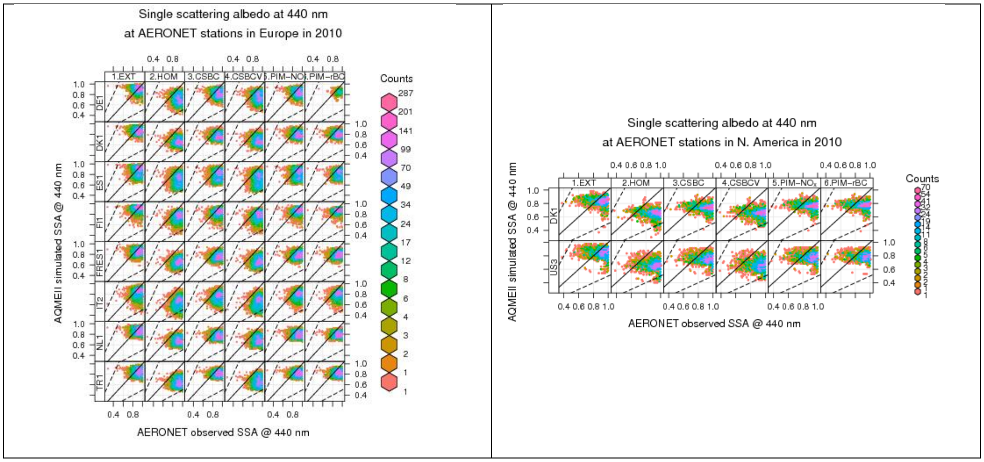

Figure S 11~~10~~. Same as Figure S 9~~Figure S 8~~, but for single scattering albedo at 440 nm ($\omega_{0,440}$).

Table S 5~~5~~. Same as Table S 3, but for single scattering albedo at 440 nm ($\omega_{0,440}$).

Label	Model	n	$\bar{O}$	$\bar{M}$	σ_O	σ_M	FAC2	MB	NMB	RMSE	r
Europe											
1.EXT	DE1	6115	0.91	0.90	0.07	0.06	1.00	0.00	0.00	0.09	-0.01
1.EXT	DK1	6115	0.91	0.87	0.07	0.05	1.00	-0.04	-0.05	0.10	-0.11
1.EXT	ES1	6115	0.91	0.87	0.07	0.08	1.00	-0.03	-0.04	0.11	0.06
1.EXT	FI1	6110	0.91	0.92	0.07	0.06	1.00	0.01	0.01	0.08	0.16
1.EXT	FRES1	6111	0.91	0.85	0.07	0.06	1.00	-0.05	-0.06	0.10	0.09
1.EXT	IT2	6115	0.91	0.84	0.07	0.09	1.00	-0.06	-0.07	0.12	0.16
1.EXT	NL1	6115	0.91	0.86	0.07	0.05	1.00	-0.04	-0.05	0.10	-0.02
1.EXT	TR1	6115	0.91	0.85	0.07	0.06	1.00	-0.05	-0.06	0.10	0.05
2.HOM	DE1	6115	0.91	0.72	0.07	0.09	1.00	-0.19	-0.21	0.22	-0.07
2.HOM	DK1	6111	0.91	0.67	0.07	0.06	1.00	-0.24	-0.26	0.25	-0.05
2.HOM	ES1	6115	0.91	0.71	0.07	0.10	1.00	-0.20	-0.22	0.23	0.06
2.HOM	FI1	6099	0.91	0.76	0.07	0.08	1.00	-0.15	-0.16	0.17	0.17
2.HOM	FRES1	6108	0.91	0.66	0.07	0.06	1.00	-0.25	-0.27	0.26	0.17
2.HOM	IT2	6115	0.91	0.65	0.07	0.10	0.96	-0.26	-0.28	0.28	0.17
2.HOM	NL1	6111	0.91	0.68	0.07	0.06	1.00	-0.22	-0.25	0.24	0.00
2.HOM	TR1	6115	0.91	0.65	0.07	0.07	1.00	-0.26	-0.28	0.27	0.05
3.CSBC	DE1	6115	0.91	0.82	0.07	0.07	1.00	-0.09	-0.10	0.13	-0.07
3.CSBC	DK1	6113	0.91	0.77	0.07	0.05	1.00	-0.14	-0.16	0.17	-0.11

Label	Model	n	$\bar{O}$	$\bar{M}$	σ_O	σ_M	FAC2	MB	NMB	RMSE	r
3.CSBC	ES1	6115	0.91	0.80	0.07	0.09	1.00	-0.11	-0.12	0.15	0.05
3.CSBC	FI1	6109	0.91	0.85	0.07	0.06	1.00	-0.06	-0.07	0.10	0.15
3.CSBC	FRES1	6109	0.91	0.76	0.07	0.06	1.00	-0.15	-0.16	0.17	0.11
3.CSBC	IT2	6115	0.91	0.76	0.07	0.08	1.00	-0.15	-0.16	0.18	0.16
3.CSBC	NL1	6113	0.91	0.77	0.07	0.05	1.00	-0.14	-0.15	0.16	-0.03
3.CSBC	TR1	6115	0.91	0.82	0.07	0.05	1.00	-0.09	-0.10	0.12	0.07
4.CSBCV	DE1	6115	0.91	0.75	0.07	0.08	1.00	-0.15	-0.17	0.19	-0.07
4.CSBCV	DK1	6111	0.91	0.68	0.07	0.07	1.00	-0.22	-0.25	0.24	-0.11
4.CSBCV	ES1	6115	0.91	0.72	0.07	0.11	1.00	-0.19	-0.21	0.23	0.05
4.CSBCV	FI1	6101	0.91	0.78	0.07	0.09	0.99	-0.13	-0.14	0.16	0.16
4.CSBCV	FRES1	6108	0.91	0.67	0.07	0.07	1.00	-0.24	-0.26	0.26	0.11
4.CSBCV	IT2	6115	0.91	0.66	0.07	0.10	0.97	-0.25	-0.27	0.27	0.17
4.CSBCV	NL1	6111	0.91	0.69	0.07	0.07	1.00	-0.22	-0.24	0.24	-0.04
4.CSBCV	TR1	6115	0.91	0.67	0.07	0.08	1.00	-0.24	-0.26	0.26	0.03
5.PIM-NOx	DE1	6111	0.91	0.84	0.07	0.06	1.00	-0.06	-0.07	0.11	-0.07
5.PIM-NOx	DK1	6113	0.91	0.79	0.07	0.05	1.00	-0.11	-0.13	0.14	-0.12
5.PIM-NOx	ES1	6020	0.91	0.82	0.07	0.08	1.00	-0.09	-0.10	0.14	0.04
5.PIM-NOx	FI1	5606	0.91	0.87	0.06	0.06	1.00	-0.04	-0.04	0.09	0.13
5.PIM-NOx	FRES1	6109	0.91	0.79	0.07	0.06	1.00	-0.12	-0.14	0.15	0.10
5.PIM-NOx	IT2	5310	0.91	0.80	0.06	0.07	1.00	-0.12	-0.13	0.14	0.18
5.PIM-NOx	NL1	6115	0.91	0.80	0.07	0.06	1.00	-0.11	-0.12	0.14	-0.05
5.PIM-NOx	TR1	6114	0.91	0.83	0.07	0.05	1.00	-0.08	-0.09	0.11	0.06
6.PIM-rBC	DE1	662	0.92	0.85	0.05	0.05	1.00	-0.08	-0.08	0.10	0.07
6.PIM-rBC	DK1	5989	0.91	0.80	0.07	0.05	1.00	-0.11	-0.12	0.14	-0.11
6.PIM-rBC	ES1	4290	0.91	0.82	0.06	0.07	1.00	-0.09	-0.10	0.13	0.08
6.PIM-rBC	FI1	4455	0.91	0.87	0.06	0.04	1.00	-0.04	-0.04	0.08	0.12
6.PIM-rBC	FRES1	6108	0.91	0.80	0.07	0.05	1.00	-0.11	-0.12	0.14	0.11
6.PIM-rBC	IT2	5018	0.91	0.80	0.06	0.07	1.00	-0.11	-0.12	0.14	0.18
6.PIM-rBC	NL1	5618	0.91	0.80	0.06	0.05	1.00	-0.11	-0.12	0.14	0.00
6.PIM-rBC	TR1	5583	0.91	0.83	0.06	0.05	1.00	-0.08	-0.09	0.11	0.06
N. America											
1.EXT	DK1	2016	0.88	0.84	0.10	0.06	1.00	-0.05	-0.05	0.13	-0.13
1.EXT	US3	2014	0.88	0.85	0.10	0.07	1.00	-0.03	-0.04	0.13	-0.10
2.HOM	DK1	2016	0.88	0.65	0.10	0.07	0.98	-0.23	-0.26	0.26	-0.15
2.HOM	US3	2014	0.88	0.66	0.10	0.07	0.98	-0.22	-0.25	0.25	-0.11
3.CSBC	DK1	2016	0.88	0.74	0.10	0.05	1.00	-0.14	-0.16	0.19	-0.14

Label	Model	n	$\bar{O}$	$\bar{M}$	σ_O	σ_M	FAC2	MB	NMB	RMSE	r
3.CSBC	US3	2014	0.88	0.76	0.10	0.06	1.00	-0.12	-0.14	0.18	-0.11
4.CSBCV	DK1	2016	0.88	0.66	0.10	0.06	0.99	-0.22	-0.25	0.26	-0.14
4.CSBCV	US3	2014	0.88	0.67	0.10	0.08	0.98	-0.22	-0.25	0.26	-0.11
5.PIM-NOx	DK1	2015	0.88	0.77	0.10	0.05	1.00	-0.12	-0.13	0.17	-0.15
5.PIM-NOx	US3	2014	0.88	0.78	0.10	0.06	1.00	-0.10	-0.11	0.16	-0.12
6.PIM-rBC	DK1	1974	0.88	0.78	0.10	0.05	1.00	-0.11	-0.12	0.16	-0.14
6.PIM-rBC	US3	1566	0.88	0.79	0.10	0.05	1.00	-0.09	-0.10	0.15	-0.08

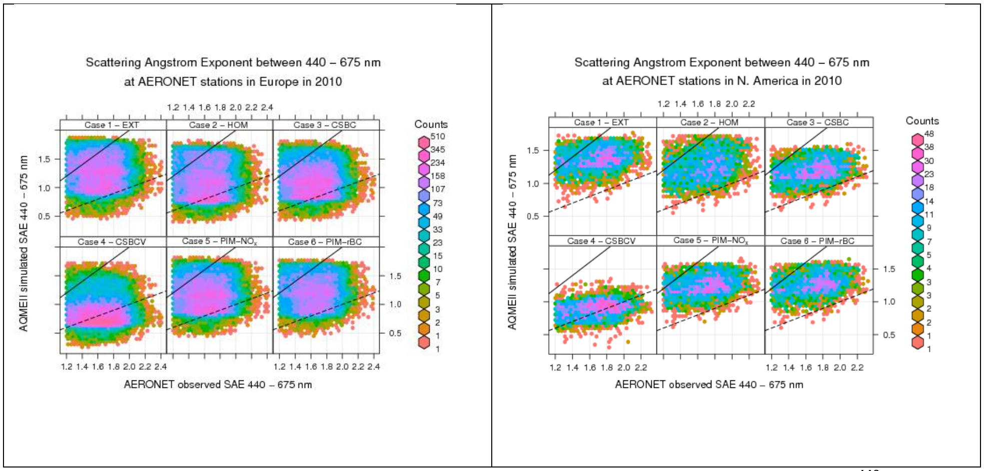

Figure S 12~~11~~. Same as Figure S 8~~Figure S 7~~, but for scattering Angstrom exponent between 440 and 675 nm (SAE_{675}^{440}).

Table S 6~~6~~. Same as Table S 1, but for scattering Angstrom exponent between 440 and 675 nm (SAE_{675}^{440}).

Europe	*n*	$\bar{O}$	$\bar{M}$	σ_O	σ_M	*FAC2*	*MB*	*NMB*	*RMSE*	*r*
1.EXT	48911	1.62	1.28	0.22	0.26	0.97	-0.34	-0.21	0.48	-0.01
2.HOM	48889	1.62	1.14	0.22	0.28	0.85	-0.48	-0.30	0.60	-0.01
3.CSBC	48904	1.62	1.14	0.22	0.25	0.90	-0.48	-0.30	0.58	0.00
4.CSBCV	48891	1.62	0.87	0.22	0.22	0.53	-0.76	-0.47	0.82	0.01
5.PIM-NOx	47498	1.62	1.18	0.22	0.25	0.93	-0.44	-0.27	0.55	0.00
6.PIM-rBC	37723	1.63	1.22	0.22	0.25	0.94	-0.41	-0.25	0.53	0.00
N. America	*n*	$\bar{O}$	$\bar{M}$	σ_O	σ_M	*FAC2*	*MB*	*NMB*	*RMSE*	*r*
1.EXT	4030	1.68	1.37	0.25	0.17	1.00	-0.31	-0.18	0.41	0.24
2.HOM	4030	1.68	1.26	0.25	0.21	0.98	-0.41	-0.25	0.50	0.27
3.CSBC	4030	1.68	1.20	0.25	0.16	0.98	-0.48	-0.28	0.54	0.29
4.CSBCV	4030	1.68	0.90	0.25	0.13	0.64	-0.78	-0.47	0.82	0.27
5.PIM-NOx	4029	1.68	1.25	0.25	0.16	0.99	-0.43	-0.26	0.50	0.26
6.PIM-rBC	3540	1.68	1.26	0.25	0.16	0.99	-0.42	-0.25	0.49	0.28

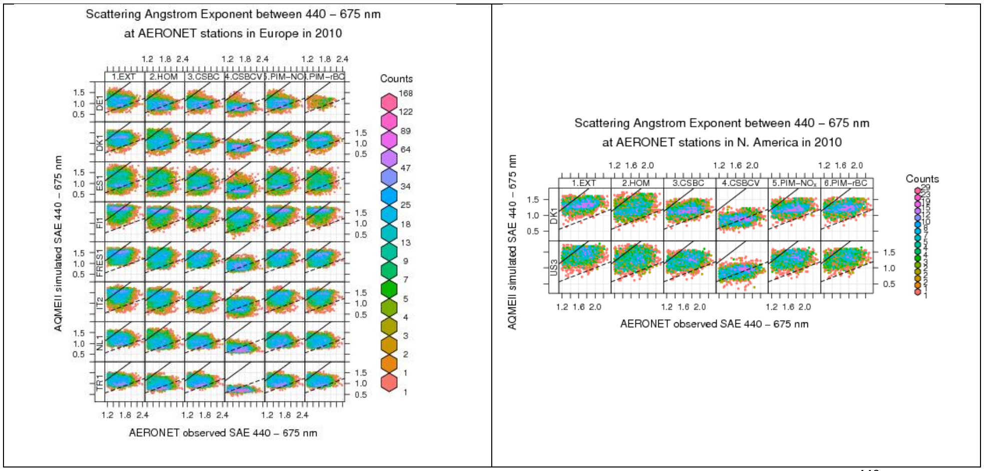

Figure S 13~~12~~. Same as Figure S 9~~Figure S 8~~, but for scattering Angstrom exponent between 440 and 675 nm (SAE_{675}^{440}).

Table S 7~~7~~. Same as Table S 3, but for scattering Angstrom exponent between 440 and 675 nm (SAE_{675}^{440}).

Label	Model	n	$\bar{O}$	$\bar{M}$	σ_O	σ_M	FAC2	MB	NMB	RMSE	r
Europe											
1.EXT	DE1	6115	1.62	1.13	0.22	0.18	0.94	-0.49	-0.30	0.57	-0.04
1.EXT	DK1	6115	1.62	1.23	0.22	0.19	0.98	-0.39	-0.24	0.48	0.00
1.EXT	ES1	6115	1.62	1.15	0.22	0.28	0.88	-0.47	-0.29	0.60	-0.06
1.EXT	FI1	6110	1.62	1.59	0.22	0.20	1.00	-0.03	-0.02	0.29	0.06
1.EXT	FRES1	6111	1.62	1.40	0.22	0.19	1.00	-0.22	-0.14	0.36	0.09
1.EXT	IT2	6115	1.62	1.40	0.22	0.22	0.98	-0.22	-0.14	0.38	0.03
1.EXT	NL1	6115	1.62	1.20	0.22	0.18	0.99	-0.42	-0.26	0.51	-0.08
1.EXT	TR1	6115	1.62	1.13	0.22	0.16	0.97	-0.49	-0.30	0.57	-0.08
2.HOM	DE1	6115	1.62	0.96	0.22	0.17	0.75	-0.67	-0.41	0.72	-0.06
2.HOM	DK1	6111	1.62	1.10	0.22	0.24	0.86	-0.52	-0.32	0.61	0.00
2.HOM	ES1	6115	1.62	1.04	0.22	0.30	0.69	-0.58	-0.36	0.70	-0.07
2.HOM	FI1	6099	1.62	1.47	0.22	0.22	0.99	-0.15	-0.09	0.34	0.05
2.HOM	FRES1	6108	1.62	1.27	0.22	0.25	0.96	-0.35	-0.22	0.48	0.08
2.HOM	IT2	6115	1.62	1.26	0.22	0.24	0.95	-0.36	-0.22	0.49	0.01
2.HOM	NL1	6111	1.62	1.09	0.22	0.23	0.86	-0.53	-0.33	0.63	-0.09
2.HOM	TR1	6115	1.62	0.96	0.22	0.18	0.71	-0.66	-0.41	0.72	-0.08
3.CSBC	DE1	6115	1.62	0.99	0.22	0.16	0.82	-0.63	-0.39	0.69	-0.05

Label	Model	n	$\bar{O}$	$\bar{M}$	σ_O	σ_M	FAC2	MB	NMB	RMSE	r
3.CSBC	DK1	6113	1.62	1.07	0.22	0.18	0.89	-0.55	-0.34	0.62	0.00
3.CSBC	ES1	6115	1.62	1.00	0.22	0.24	0.72	-0.62	-0.38	0.70	-0.06
3.CSBC	FI1	6109	1.62	1.47	0.22	0.22	0.99	-0.15	-0.10	0.34	0.06
3.CSBC	FRES1	6109	1.62	1.23	0.22	0.20	0.98	-0.39	-0.24	0.48	0.11
3.CSBC	IT2	6115	1.62	1.22	0.22	0.22	0.95	-0.40	-0.25	0.50	0.06
3.CSBC	NL1	6113	1.62	1.04	0.22	0.17	0.86	-0.58	-0.36	0.64	-0.07
3.CSBC	TR1	6115	1.62	1.10	0.22	0.16	0.95	-0.52	-0.32	0.59	-0.06
4.CSBCV	DE1	6115	1.62	0.83	0.22	0.13	0.52	-0.79	-0.49	0.83	-0.03
4.CSBCV	DK1	6111	1.62	0.83	0.22	0.12	0.52	-0.79	-0.49	0.83	-0.05
4.CSBCV	ES1	6115	1.62	0.75	0.22	0.18	0.34	-0.87	-0.54	0.92	-0.04
4.CSBCV	FI1	6101	1.62	1.23	0.22	0.24	0.92	-0.40	-0.24	0.51	0.06
4.CSBCV	FRES1	6108	1.62	0.92	0.22	0.14	0.74	-0.70	-0.43	0.74	0.13
4.CSBCV	IT2	6115	1.62	0.88	0.22	0.20	0.61	-0.74	-0.46	0.79	0.09
4.CSBCV	NL1	6111	1.62	0.77	0.22	0.11	0.36	-0.85	-0.53	0.89	-0.10
4.CSBCV	TR1	6115	1.62	0.71	0.22	0.06	0.23	-0.91	-0.56	0.94	-0.10
5.PIM-NOx	DE1	6111	1.62	1.03	0.22	0.16	0.87	-0.59	-0.37	0.66	-0.05
5.PIM-NOx	DK1	6113	1.62	1.12	0.22	0.18	0.93	-0.50	-0.31	0.57	-0.01
5.PIM-NOx	ES1	6020	1.62	1.05	0.22	0.25	0.79	-0.57	-0.35	0.67	-0.06
5.PIM-NOx	FI1	5606	1.63	1.52	0.22	0.20	1.00	-0.11	-0.07	0.31	0.04
5.PIM-NOx	FRES1	6109	1.62	1.28	0.22	0.19	0.99	-0.35	-0.21	0.44	0.10
5.PIM-NOx	IT2	5310	1.63	1.30	0.22	0.21	0.97	-0.33	-0.20	0.45	0.04
5.PIM-NOx	NL1	6115	1.62	1.09	0.22	0.17	0.93	-0.53	-0.32	0.60	-0.08
5.PIM-NOx	TR1	6114	1.62	1.10	0.22	0.16	0.96	-0.52	-0.32	0.59	-0.07
6.PIM-rBC	DE1	662	1.68	1.06	0.21	0.15	0.89	-0.62	-0.37	0.67	0.11
6.PIM-rBC	DK1	5989	1.62	1.14	0.22	0.18	0.93	-0.49	-0.30	0.57	0.00
6.PIM-rBC	ES1	4290	1.63	1.07	0.22	0.26	0.79	-0.55	-0.34	0.66	-0.07
6.PIM-rBC	FI1	4455	1.63	1.54	0.22	0.18	1.00	-0.09	-0.06	0.29	0.04
6.PIM-rBC	FRES1	6108	1.62	1.30	0.22	0.20	0.99	-0.33	-0.20	0.43	0.10
6.PIM-rBC	IT2	5018	1.63	1.32	0.22	0.22	0.97	-0.32	-0.19	0.44	0.04
6.PIM-rBC	NL1	5618	1.62	1.11	0.22	0.18	0.92	-0.52	-0.32	0.60	-0.07
6.PIM-rBC	TR1	5583	1.63	1.11	0.22	0.16	0.96	-0.51	-0.32	0.59	-0.08
N. America											
1.EXT	DK1	2016	1.68	1.34	0.25	0.14	1.00	-0.34	-0.20	0.42	0.27
1.EXT	US3	2014	1.68	1.40	0.25	0.18	0.99	-0.28	-0.17	0.39	0.22
2.HOM	DK1	2016	1.68	1.24	0.25	0.21	0.98	-0.44	-0.26	0.51	0.30
2.HOM	US3	2014	1.68	1.29	0.25	0.22	0.97	-0.39	-0.23	0.49	0.24

Label	Model	n	$\bar{O}$	$\bar{M}$	σ_O	σ_M	FAC2	MB	NMB	RMSE	r
3.CSBC	DK1	2016	1.68	1.17	0.25	0.14	0.98	-0.50	-0.30	0.56	0.32
3.CSBC	US3	2014	1.68	1.23	0.25	0.17	0.98	-0.45	-0.27	0.52	0.28
4.CSBCV	DK1	2016	1.68	0.89	0.25	0.12	0.62	-0.78	-0.47	0.82	0.24
4.CSBCV	US3	2014	1.68	0.90	0.25	0.14	0.66	-0.78	-0.46	0.82	0.29
5.PIM-NOx	DK1	2015	1.68	1.22	0.25	0.14	0.99	-0.46	-0.27	0.52	0.29
5.PIM-NOx	US3	2014	1.68	1.28	0.25	0.17	0.99	-0.40	-0.24	0.48	0.25
6.PIM-rBC	DK1	1974	1.68	1.24	0.25	0.14	0.99	-0.44	-0.26	0.51	0.28
6.PIM-rBC	US3	1566	1.69	1.30	0.25	0.18	0.99	-0.39	-0.23	0.47	0.28

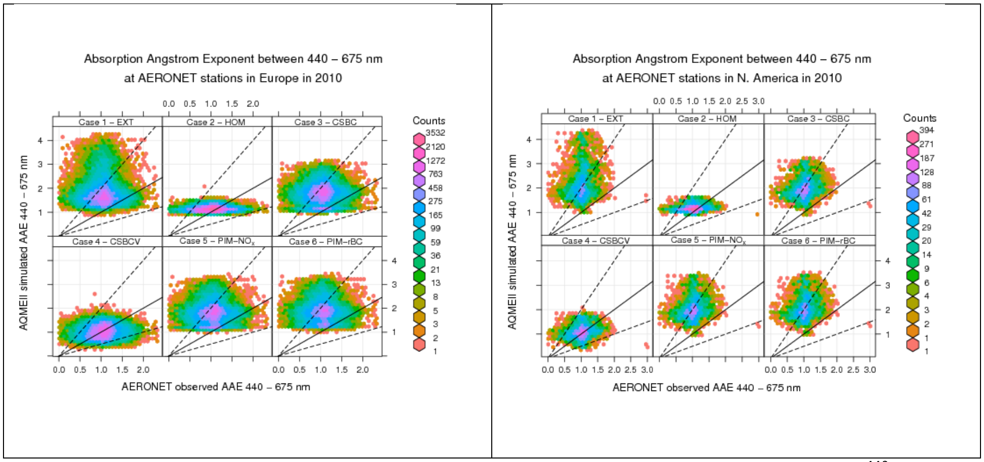

Figure S 14~~13~~. Same as Figure S 8~~Figure S 7~~, but for absorption Angstrom exponent between 440 and 675 nm (AAE_{675}^{440}).

Table S 8~~8~~. Same as Table S 1

Europe	*n*	$\bar{O}$	$\bar{M}$	σ_O	σ_M	*FAC2*	*MB*	*NMB*	*RMSE*	*r*
1.EXT	48911	1.04	1.85	0.29	0.52	0.66	0.81	0.78	1.00	0.01
2.HOM	48889	1.04	1.16	0.29	0.10	0.94	0.12	0.12	0.32	0.06
3.CSBC	48904	1.04	1.79	0.29	0.39	0.68	0.75	0.73	0.89	0.05
4.CSBCV	48891	1.04	1.04	0.29	0.25	0.93	0.01	0.01	0.36	0.10
5.PIM-NOx	47498	1.04	1.81	0.29	0.40	0.68	0.77	0.74	0.91	0.04
6.PIM-rBC	37723	1.04	1.87	0.29	0.41	0.64	0.83	0.80	0.97	0.02
N. America	*n*	$\bar{O}$	$\bar{M}$	σ_O	σ_M	*FAC2*	*MB*	*NMB*	*RMSE*	*r*
1.EXT	4030	1.02	2.28	0.29	0.71	0.42	1.26	1.23	1.45	0.17
2.HOM	4030	1.02	1.20	0.29	0.13	0.93	0.18	0.18	0.35	0.16
3.CSBC	4030	1.02	2.03	0.29	0.41	0.52	1.01	0.99	1.11	0.19
4.CSBCV	4030	1.02	1.12	0.29	0.28	0.92	0.10	0.10	0.38	0.18
5.PIM-NOx	4029	1.02	2.08	0.29	0.45	0.50	1.05	1.03	1.16	0.18
6.PIM-rBC	3540	1.03	2.12	0.29	0.48	0.48	1.09	1.06	1.21	0.18

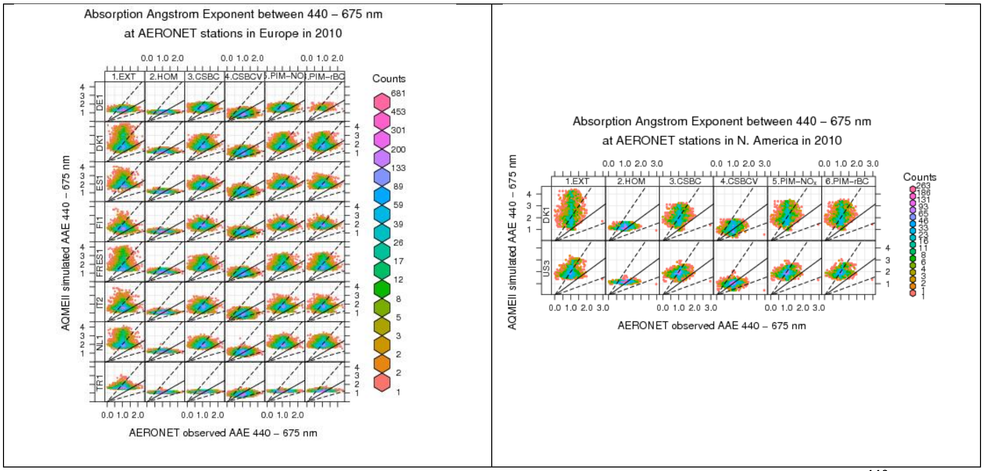

Figure S 1514. Same as Figure S 9Figure S 8, but for absorption Angstrom exponent between 440 and 675 nm (AAE_{675}^{440}).

Table S 99. Same as Table S 2, but for absorption Angstrom exponent between 440 and 675 nm (AAE_{675}^{440}).

Label	Model	n	$\bar{O}$	$\bar{M}$	σ_O	σ_M	FAC2	MB	NMB	RMSE	r
Europe											
1.EXT	DE1	6115	1.04	1.40	0.29	0.15	0.89	0.36	0.35	0.47	0.14
1.EXT	DK1	6115	1.04	2.09	0.29	0.67	0.55	1.06	1.02	1.28	0.05
1.EXT	ES1	6115	1.04	2.04	0.29	0.42	0.55	1.00	0.97	1.12	0.00
1.EXT	FI1	6110	1.04	1.63	0.29	0.32	0.78	0.59	0.57	0.74	-0.03
1.EXT	FRES1	6111	1.04	1.94	0.29	0.66	0.62	0.91	0.88	1.16	0.01
1.EXT	IT2	6115	1.04	1.74	0.29	0.38	0.72	0.70	0.68	0.85	-0.01
1.EXT	NL1	6115	1.04	2.24	0.29	0.51	0.45	1.21	1.16	1.34	0.01
1.EXT	TR1	6115	1.04	1.70	0.29	0.18	0.76	0.66	0.64	0.74	0.02
2.HOM	DE1	6115	1.04	1.10	0.29	0.07	0.95	0.07	0.06	0.29	0.17
2.HOM	DK1	6111	1.04	1.18	0.29	0.11	0.93	0.14	0.14	0.33	0.06
2.HOM	ES1	6115	1.04	1.17	0.29	0.08	0.94	0.13	0.13	0.32	0.07
2.HOM	FI1	6099	1.03	1.22	0.29	0.09	0.93	0.19	0.18	0.35	0.00
2.HOM	FRES1	6108	1.04	1.16	0.29	0.11	0.94	0.13	0.12	0.33	0.06
2.HOM	IT2	6115	1.04	1.12	0.29	0.10	0.95	0.09	0.08	0.31	0.06
2.HOM	NL1	6111	1.04	1.19	0.29	0.09	0.93	0.16	0.15	0.34	0.05
2.HOM	TR1	6115	1.04	1.10	0.29	0.05	0.95	0.06	0.06	0.29	0.19
3.CSBC	DE1	6115	1.04	1.62	0.29	0.23	0.81	0.58	0.56	0.67	0.18

Label	Model	n	$\bar{O}$	$\bar{M}$	σ_O	σ_M	FAC2	MB	NMB	RMSE	r
3.CSBC	DK1	6113	1.04	1.94	0.29	0.36	0.61	0.91	0.88	1.01	0.08
3.CSBC	ES1	6115	1.04	1.96	0.29	0.27	0.60	0.92	0.89	1.00	0.07
3.CSBC	FI1	6109	1.04	1.94	0.29	0.24	0.61	0.90	0.87	0.98	-0.01
3.CSBC	FRES1	6109	1.04	1.88	0.29	0.38	0.64	0.84	0.82	0.96	0.05
3.CSBC	IT2	6115	1.04	1.77	0.29	0.32	0.71	0.73	0.71	0.84	0.05
3.CSBC	NL1	6113	1.04	2.04	0.29	0.27	0.54	1.01	0.97	1.08	0.07
3.CSBC	TR1	6115	1.04	1.15	0.29	0.06	0.94	0.12	0.12	0.31	0.23
4.CSBCV	DE1	6115	1.04	0.91	0.29	0.23	0.92	-0.12	-0.12	0.36	0.18
4.CSBCV	DK1	6111	1.04	1.10	0.29	0.25	0.94	0.07	0.06	0.37	0.10
4.CSBCV	ES1	6115	1.04	1.15	0.29	0.27	0.92	0.12	0.11	0.39	0.09
4.CSBCV	FI1	6101	1.03	1.15	0.29	0.22	0.93	0.12	0.11	0.38	0.02
4.CSBCV	FRES1	6108	1.04	1.01	0.29	0.24	0.93	-0.02	-0.02	0.36	0.07
4.CSBCV	IT2	6115	1.04	0.94	0.29	0.25	0.89	-0.09	-0.09	0.38	0.08
4.CSBCV	NL1	6111	1.04	1.16	0.29	0.21	0.93	0.12	0.12	0.36	0.12
4.CSBCV	TR1	6115	1.04	0.92	0.29	0.16	0.95	-0.12	-0.11	0.32	0.22
5.PIM-NOx	DE1	6111	1.04	1.59	0.29	0.21	0.82	0.55	0.53	0.64	0.18
5.PIM-NOx	DK1	6113	1.04	1.97	0.29	0.41	0.59	0.93	0.90	1.05	0.07
5.PIM-NOx	ES1	6020	1.04	1.98	0.29	0.27	0.59	0.94	0.91	1.02	0.05
5.PIM-NOx	FI1	5606	1.04	1.91	0.29	0.23	0.63	0.87	0.84	0.95	-0.03
5.PIM-NOx	FRES1	6109	1.04	1.89	0.29	0.42	0.62	0.86	0.83	1.00	0.04
5.PIM-NOx	IT2	5310	1.04	1.79	0.29	0.32	0.70	0.75	0.72	0.86	0.02
5.PIM-NOx	NL1	6115	1.04	2.08	0.29	0.31	0.51	1.05	1.01	1.13	0.05
5.PIM-NOx	TR1	6114	1.04	1.24	0.29	0.05	0.93	0.20	0.19	0.35	0.19
6.PIM-rBC	DE1	662	1.05	1.62	0.27	0.19	0.81	0.57	0.55	0.66	0.11
6.PIM-rBC	DK1	5989	1.04	1.99	0.29	0.43	0.59	0.95	0.92	1.08	0.07
6.PIM-rBC	ES1	4290	1.04	2.06	0.29	0.28	0.53	1.02	0.98	1.09	0.01
6.PIM-rBC	FI1	4455	1.04	1.97	0.29	0.22	0.60	0.93	0.89	1.00	-0.04
6.PIM-rBC	FRES1	6108	1.04	1.91	0.29	0.44	0.62	0.87	0.84	1.02	0.04
6.PIM-rBC	IT2	5018	1.05	1.80	0.29	0.32	0.69	0.75	0.72	0.87	0.01
6.PIM-rBC	NL1	5618	1.04	2.12	0.29	0.33	0.50	1.08	1.04	1.16	0.03
6.PIM-rBC	TR1	5583	1.04	1.32	0.29	0.06	0.91	0.28	0.27	0.40	0.05
N. America											
1.EXT	DK1	2016	1.02	2.49	0.29	0.86	0.37	1.47	1.44	1.71	0.17
1.EXT	US3	2014	1.02	2.06	0.29	0.41	0.46	1.04	1.02	1.13	0.23
2.HOM	DK1	2016	1.02	1.24	0.29	0.16	0.92	0.22	0.21	0.37	0.17
2.HOM	US3	2014	1.02	1.17	0.29	0.08	0.93	0.15	0.15	0.32	0.20

Label	Model	n	$\bar{O}$	$\bar{M}$	σ_O	σ_M	FAC2	MB	NMB	RMSE	r
3.CSBC	DK1	2016	1.02	2.11	0.29	0.50	0.48	1.09	1.07	1.21	0.18
3.CSBC	US3	2014	1.02	1.94	0.29	0.26	0.57	0.92	0.90	0.98	0.24
4.CSBCV	DK1	2016	1.02	1.19	0.29	0.33	0.90	0.17	0.17	0.43	0.20
4.CSBCV	US3	2014	1.02	1.05	0.29	0.19	0.93	0.03	0.03	0.32	0.17
5.PIM-NOx	DK1	2015	1.02	2.18	0.29	0.56	0.45	1.16	1.13	1.30	0.17
5.PIM-NOx	US3	2014	1.02	1.97	0.29	0.28	0.55	0.95	0.93	1.01	0.24
6.PIM-rBC	DK1	1974	1.02	2.21	0.29	0.57	0.44	1.18	1.16	1.33	0.17
6.PIM-rBC	US3	1566	1.04	2.01	0.29	0.29	0.53	0.97	0.94	1.04	0.26
